# What is the evidence on indirect drivers of biodiversity loss worldwide? A systematic map protocol

**DOI:** 10.1371/journal.pone.0341928

**Published:** 2026-03-06

**Authors:** Federico Grossi, Daniel I. Avila-Ortega, Anne-Sophie Crépin, Elina A. Virtanen, Enrico Di Minin

**Affiliations:** 1 Helsinki Lab of Interdisciplinary Conservation Science, Department of Geosciences and Geography, University of Helsinki, Helsinki, Finland; 2 Helsinki Institute of Sustainability Science (HELSUS), University of Helsinki, Helsinki, Finland; 3 Stockholm Resilience Centre, Stockholm University, Stockholm, Sweden; 4 Global Economic Dynamics and the Biosphere Programme, Royal Swedish Academy of Sciences; 5 Beijer Institute of Ecological Economics, Royal Swedish Academy of Sciences, Stockholm, Sweden; 6 Finnish Natural History Museum (LUOMUS), University of Helsinki, Helsinki, Finland; 7 Finnish Environment Institute (Syke), Helsinki, Finland; 8 Centre for Sustainability Transitions, Stellenbosch University, Stellenbosch, South Africa; USDA Forest Service Southern Research Station, UNITED STATES OF AMERICA

## Abstract

An estimated 1 million species of animals and plants are threatened with extinction. While much research has concentrated on direct drivers (e.g., land use change) of biodiversity loss, understanding the indirect (e.g., economic, demographic, and institutional, etc.) drivers triggering these direct drivers is a research priority. Systematic reviews of literature mapping the links between indirect and direct drivers and where, when, how, and what biodiversity they affect are missing. The primary research question of this systematic map will be: *What is the evidence on indirect drivers of biodiversity loss worldwide*? Eligible studies must include information on the links between direct and indirect drivers and how they affect biodiversity. Outcomes will be categorized by species, taxonomic groups, ecological levels of organization, geographical scope, and both direct and indirect drivers of biodiversity loss. We will implement a thorough and repeatable three-stage screening process through peer-reviewed articles, and a searchable database will be made publicly accessible as an interactive online resource. This systematic map will help identify knowledge gaps and enhance our understanding of the underlying causes of biodiversity loss.

## Introduction

Approximately 1 million animal and plant species are currently threatened with extinction, many within the coming decades [1]. From 1970 to 2006, global indicators such as species population trends, threatened species, habitat extent and condition, and community composition have consistently deteriorated, while indicators of human pressures on biodiversity showed an average increase of 50% over the same period [2]. On land, approximately 75% of terrestrial environments have been significantly altered, primarily due to agriculture, urban expansion, and infrastructure development [1]. Similarly, 77% of terrestrial land (excluding Antarctica) is subject to measurable human pressure, with only 23% remaining relatively undisturbed [3]. Further analyses show that over 50% of terrestrial land is now moderately to highly modified, posing substantial risks to biodiversity, connectivity, and ecological resilience [4]. In marine systems, 97.7% of marine areas are affected by human activities, with 66% experiencing moderate to high cumulative pressures, primarily from climate change, fishing, and coastal development [5]. By 2019, impacts were increasing in 59% of ocean areas, with climate-related stressors emerging as the dominant pressure [6]. More recent analyses confirm this trend: over 21,000 marine species are now exposed to cumulative risks [7], and approximately 84% of the world’s coral reefs experienced bleaching-level heat stress during the 2023–2025 global bleaching event [8]. These changes are compounded by ocean acidification nearing critical thresholds [9], highlighting an accelerating crisis for marine ecosystems. Overall, the escalating loss of biodiversity across ecosystems is undermining the contributions nature provides to people and directly affecting human well-being [10,11].

Direct drivers of biodiversity loss, also known as threats, represent “*the proximate human activities or processes that have caused, are causing, or may cause the destruction, degradation, and/or impairment of biodiversity targets*” [12]. The main drivers of biodiversity loss are land and sea use change, direct exploitation, climate change, pollution, and invasive alien species [13]. The International Union for the Conservation of Nature (IUCN) has further classified these main drivers into twelve broad sub-categories [12]. For example, the land and sea use change drivers are further classified into ‘residential and commercial development’, ‘agriculture and aquaculture’, ‘energy production and mining’, and ‘transportation and service corridors’ sub-categories. While the conservation science literature covers direct drivers extensively [e.g., 13–17], the indirect drivers of biodiversity loss that underlie and shape the extent, severity, and combination of direct drivers that operate in a given place are still poorly studied.

Indirect drivers are factors that act in a diffuse manner, shaping and influencing direct drivers as well as other indirect drivers [18]. They can be broadly classified into (i) demographic and socio-cultural; (ii) economic and technological; (iii) institutions and governance; and (iv) conflicts and epidemics [1,10]. Indirect drivers typically act in combination, spanning multiple scales and varying distances from the affected area. They range from global influences (such as markets, commodity prices, and consumption trends) to national and regional factors (including demographic shifts, migration, domestic markets, policies, governance, and cultural or technological changes) and local conditions (like poverty and economic opportunities) [19]**.** Socio-economic and demographic patterns are key in shaping consumption patterns, which can then have subsequent negative impacts on biodiversity via land use change transformations [20]**.** Technological innovations and governance can contribute to biodiversity loss through the increased demand for construction space [21,22] and sourcing raw materials in countries where the rule of law is the weakest [23]. Hence, unveiling the global interconnected network of indirect drivers is central to bending the curve of biodiversity loss [3,24–26]. It is also a research priority to understand how these indirect drivers are underpinned by diverse values of nature and human behavior [13].

Behind direct and indirect drivers of biodiversity loss lie deep-rooted and interconnected patterns, known as underlying causes, that shape, influence and reinforce biodiversity loss and nature’s decline [27]**.** These causes reflect deeply embedded worldviews and institutional norms that shape human-nature relations and influence systemic patterns of behavior. The IPBES Transformative Change Assessment [27] identifies three principal underlying causes: (1) a pervasive disconnection from, and perceived domination over, nature and marginalized peoples, which legitimizes extractive and exploitative practices; (2) the concentration of power and wealth, which facilitates disproportionate resource consumption and enables resistance to systemic transformation; and (3) the prioritization of short-term, individualistic, and material objectives, often institutionalized through economic frameworks that emphasize GDP growth at the expense of long-term ecological and social sustainability. While the role of underlying causes in shaping biodiversity outcomes is acknowledged, a detailed examination of these foundational societal and cultural drivers lies beyond the scope of this manuscript. This study will instead focus on the more proximate direct and indirect drivers of biodiversity loss, which are more readily observable and actionable within the framework of the present analysis.

Recent studies have mapped threats to species [16] and human pressures on biodiversity [14], or reviewed present [13] and future [15,17] threats to biodiversity. However, the authors could find no previous systematic mapping study or review focusing on the indirect drivers of biodiversity loss. This systematic map protocol outlines the methodology for identifying such literature. We aim to categorize indirect drivers following Díaz et al. [13] and distinguish their geographic origins from the areas they impact. To assess the spatial connection between direct and indirect drivers of biodiversity loss, we will follow the telecoupling framework [28] that tracks complex interactions between human and natural systems across distances. By cross-linking information on indirect drivers, direct drivers, and impacted organisms, we aim to better understand the chain of causation from broad economic, demographic, and technological factors to specific biodiversity outcomes and trends. Relevant information concerning all taxonomic groups, ecoregions, habitats, and ecosystems will be included. We will develop a static, searchable, online database to host the full evidence base. This database will include metadata on each included study, such as geographic focus, indirect and direct drivers examined, taxonomic groups, and ecological levels of organization (from populations to biomes), and will be accessible through a web-based graphical user interface. The aim is to enhance the usability, transparency, and long-term value of the systematic map by allowing researchers, policymakers, and other stakeholders to explore and filter the evidence according to their needs. This systematic mapping protocol builds on recent work to promote consistency and continuity across databases [16]**.** Overall, the results will increase our understanding of how indirect pressures can be systematically documented and benefit the conservation science and practice community. The primary research question for this systematic mapping study will be: What is the evidence on the indirect drivers of biodiversity loss worldwide? A targeted data extraction strategy will allow the creation of a user searchable database to answer the following secondary research questions:

1. How many studies explicitly identify and describe indirect drivers in the context of direct drivers of biodiversity loss? Where have these studies been conducted?
2. How have indirect drivers of biodiversity loss been defined, operationalized, and analyzed in the existing literature?
3. Which categories of indirect drivers are most frequently studied?
4. Which taxonomic groups and ecological levels are most commonly studied when indirect drivers are examined in relation to direct drivers of biodiversity loss?
5. How often and where do direct and indirect drivers of biodiversity loss overlap geographically, and what taxonomic groups are involved?
6. What knowledge gaps are there, and what do they entail?

The study has the potential to systematically identify whether and how indirect and direct drivers contribute to biodiversity loss across different locations, by mapping these relationships at multiple spatial scales. Specifically, it will be possible to identify what biodiversity is impacted by which indirect drivers, where these are located, and what their nature is. It will also be possible to understand how knowledge concerning pressures on biodiversity acting at a distance is collected and discussed in scientific literature.

## Materials and methods

This systematic mapping exercise will adhere to the guidelines set out by the Preferred Reporting Items for Systematic Review and Meta-Analysis Protocols (PRISMA-P) [29] (**S1 File**) and complemented with the Guidelines and Standards for Evidence Synthesis in Environmental Management from the Collaboration for Environmental Evidence [30], incorporating the Reporting standards for Systematic Evidence Syntheses (ROSES) [31] (**S1 Table**).

### Search plan

The search plan included a scoping phase during which we conducted a string search on Scopus using the following terms: (systematic mapping OR systematic literature review OR systematic review) AND (indirect driver*) AND (biodiversity loss) (**S2 Table**); however, this search yielded no relevant results. A subsequent search specifically targeting indirect drivers of biodiversity loss, or indirect threats, resulted in fewer than 400 articles, indicating a significant underrepresentation of knowledge regarding drivers of biodiversity loss and their remote sources. For this second preliminary search, we used the following search string: (indirect) AND (threat* OR driver* OR pressure*) AND (biodiversity OR biodiversity loss). Consequently, we opted to examine documents addressing direct drivers of biodiversity loss, assessing whether the authors acknowledged the presence of indirect sources in terms of typology and spatial scale. Because our approach emphasizes direct drivers over indirect ones, we recognize that authors of eligible papers might briefly mention indirect drivers to provide context for a direct driver of biodiversity loss. To facilitate the mapping process, we established a benchmark list of fifteen articles to guide our search and evaluate the comprehensiveness of our findings (**S2 File**). These fifteen articles were selected to be representative of a broad spectrum of research methodologies, geographic scales, ecoregions, ecological levels of organization, and direct and indirect drivers. We extracted official and unofficial keywords from the titles and abstracts of these articles and organized them into thematic terms. Search strings were then run on OpenAlex and the resulting retrieved documents were compared (**S2 Table**). OpenAlex is an open-access bibliographic catalogue of scientific articles, authors, institutions, and other bibliographic metadata records including more than 250 million works such as books, datasets, and journal articles [32]. It has been compared with databases behind a paywall (e.g., Scopus, Web of Science) and search engines (e.g., Google Scholar, Semantic Scholar), showing a good degree of article coverage, availability, and metadata records [33,34]. Candidate search strings were tested based on the number of search terms and retrieved documents. Each search attempt was evaluated for comprehensiveness based on its capacity to yield the full list of fifteen benchmark articles (**S2 File**). Because we will bulk download an extensive literature base and filter it during successive stages, we choose the search string with the maximum combination of relevant broad words, which is the following:

(pressures OR threats OR footprint OR risk OR drivers OR stress OR impacts OR loss) AND (species OR ecosystems OR wildlife OR habitat) AND (hotspot OR map OR spatial OR direct OR indirect OR feedback OR long-distance OR human OR anthropogenic OR actions) AND (biodiversity OR conservation)

We will restrict sources to literature published between 1999 and 2024 published in English, Spanish, French, and Italian due to the authors’ fluency in these languages, which ensures accurate comprehension and analysis. Focusing our systematic literature mapping on the past 25 years will ensure relevance by incorporating current theories, methodologies, and technologies, highlighting contemporary trends and gaps, and making the review process more manageable. Firstly, we will use the IUCN Red List threat classification scheme [12] to categorize existing direct drivers of biodiversity loss. Secondly, we will manually compile relevant information on impacted organisms, taxonomic groups, ecological levels of organization, countries, and ecoregions. Thirdly, we will manually collect data on indirect drivers when explicitly mentioned (i.e., observed or recorded), hypothesized (i.e., modeled on an existing and observed threat), or referred to from another source (i.e., external citation). The outcome to be assessed will be the presence of an explicit term indicating indirect drivers of anthropogenic direct drivers of biodiversity loss. We will classify human-driven indirect factors following Díaz et al. [13]. For our research purposes, we will consider the terms *‘indirect drivers (of biodiversity loss)*’ and *‘indirect threats* (*to biodiversity)’* as synonymous. Similarly, we will also treat as synonyms the terms related to ‘*direct drivers (of biodiversity loss)*’, namely ‘*direct threats (to biodiversity)’*, ‘*threats*’, ‘*IUCN threat categories*’, ‘*sources of stress*’, and ‘*proximate pressures*’. When possible, we will investigate the spatial source of indirect drivers differentiating between those acting at the same spatial scale as direct drivers and those that are not.

We anticipate that data collection will be finalized between September and October 2026. This timeline accounts for the comprehensive search and screening process required for a systematic map. Following the completion of data collection, we expect to analyze the data and prepare the results for publication. The results will likely be available between January and February 2027. This period includes time for data synthesis, interpretation, and manuscript preparation. We acknowledge that unforeseen delays may arise during the systematic mapping process. Potential challenges include slower-than-anticipated full-text screening due to limited team availability, a larger-than-expected volume of search results, and difficulties in obtaining abstracts or full-text articles. To mitigate these risks, we have incorporated buffer periods into the timeline, we will conduct regular team check-ins to monitor progress, and we will reassign tasks or adjust workloads as necessary. In addition, we are using automation tools to support screening and data management, which we anticipate will improve efficiency and help prevent process bottlenecks. All data generated during the systematic mapping process, including the bibliographic database, screening stages, and coding extraction strategy, will be made publicly available via Zenodo, in line with open science principles. Copyrighted materials such as full-text PDFs will not be shared publicly but will be referenced appropriately. We emphasize both rigorous data stewardship and transparent authorship practices. Detailed records of data collection, coding, and extraction strategies will be maintained and made accessible to ensure reproducibility. Sensitive information will be anonymized in accordance with ethical guidelines. Automation tools used in screening and data management will be validated through systematic manual checks, and all updates to the dataset or methodology will be carefully documented to maintain a clear audit trail.

### Bibliographic databases and data management

A structured full-text search will be conducted exclusively on OpenAlex [32], focusing on academic literature that includes peer-reviewed research articles and book chapters (i.e., “journal article” and “book chapter” types). Given the extensive nature of the literature base, we will exclude additional sources such as organizational websites or grey literature. The collected data will undergo filtering at various stages to minimize noise in the literature base. First, we will concentrate on primary research published in English, Spanish, French, or Italian, from 1999 to 2024. We will not consider retracted articles, non-article types, articles missing reference lists. duplicate records, and articles without a DOI. Second, we will eliminate all duplicates. Additionally, the initial benchmark list has been expanded to include 182 papers known to be relevant to the authors, which will be utilized to train an automated code for filtering the literature retrieved from OpenAlex (**S3 Table**) [35]. All 182 papers were manually screened at the title and abstract level and confirmed as eligible for inclusion. This curated set constitutes the positive training dataset used to calibrate and evaluate the automated screening that will be applied to the larger corpus retrieved from OpenAlex. The benchmark articles were selected to be representative of a broad spectrum of research methodologies, geographic scales, taxonomic groups, ecological levels of organization, and direct and indirect drivers of biodiversity loss.

**Fig 1:**
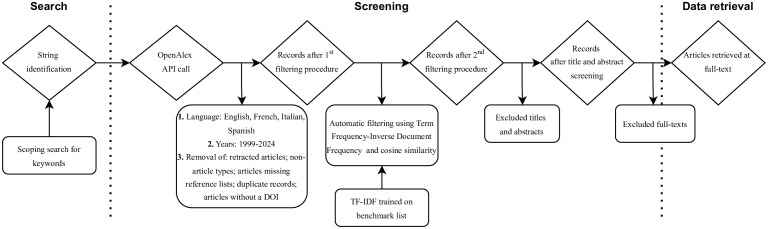
Workflow for literature search, screening, and data retrieval. Flow of articles through the screening process, following ROSES reporting standards for systematic evidence synthesis [31] and incorporating machine learning–assisted screening (adapted from Avila-Ortega et al.[35]), including OpenAlex retrieval, metadata filtering, TF-IDF and cosine similarity filtering, title/abstract screening, and full-text selection.

Filtered articles will be downloaded using the PyAlex Python library [36]. In Python (v3.7.12), we will apply the following exclusion criteria: (i) retracted articles, (ii) non-article types (e.g., editorials, datasets, reports, books, letters), (iii) articles missing reference lists, (iv) duplicate records, and (v) articles without a DOI will be excluded. Title and abstract screening will be conducted using Natural Language Processing (NLP), applying Term Frequency–Inverse Document Frequency (TF-IDF) vectorization [37–39] and cosine similarity [40]. First, TF-IDF will be trained on the benchmark article titles and applied to all article titles, using a cosine similarity threshold of 15%. For records lacking abstracts, we will attempt automated extraction using tools such as citationchaser [41] and crossref-commons [42], and, if unsuccessful, we will proceed with manual recovery of available abstracts. A second TF-IDF model will then be trained on benchmark abstracts, using a 13% cosine similarity threshold for screening remaining records. If the reported set thresholds do not fully capture the benchmark articles, they will be adjusted accordingly. Finally, full-text articles will be manually screened for inclusion. All data processing will be conducted using dask v2022.2.0 [43], pandas v1.3.5 [44], numpy v1.21.6 [45], nltk v3.8.1 [46], and scikit-learn v1.0.2 [47]. All scripts and workflows will be made available in the project’s GitHub repository **(**Fig 1**)**. After the NLP-assisted title and abstract screening, each review will be performed by human reviewers rather than deep-learning models. The rationale is to ensure interpretative accuracy and maintain transparency in classification decisions. While AI-based semantic approaches, such as embeddings or transformer models, can facilitate or fully automate the screening process, these would inevitably introduce complexity and potential bias that we aim to avoid for this study.

### Article screening and study inclusion criteria

#### Screening strategy.

We will undertake a three-stage screening process (title, abstract, and full-text). A TF-IDF model trained to narrow down the corpus based on (dis)similarities against a collection of benchmark articles will be used for the screening and filtering stages of titles and abstracts. Manual full-text screening will take place using revtools [48,49], a web-based tool used to assist screening and selection of studies during systematic reviews, systematic maps, and other evidence synthesis processes*.* We will use revtools to support the manual title and abstract screening stage of the systematic map, allowing for independent, blinded screening by all reviewers and providing an efficient process for identifying and resolving disagreements. All rejected papers and the justifications for their exclusion during the full-text phase will be recorded for transparency.

#### Inclusion criteria.

To ensure consistency with recent systematic mapping exercises in conservation science, we are adapting Ridley et al. [16]’s data extraction strategy to identify indirect drivers of biodiversity loss within the literature on direct species threats.

1. Eligible population: This study includes all organisms globally, covering all countries and ecoregions (terrestrial, marine, and freshwater), and at different selected ecological levels (populations, communities, ecosystems, habitats, and biomes). To be incorporated into the analysis, studies must present relevant information at any of these selected ecological levels. This evidence may rely on species’ known range, and specific designated areas prioritized for biodiversity conservation, such as Key Biodiversity Areas [50] or Biodiversity Hotspots [51,52].
2. Eligible outcome: The evidenced presence of anthropogenic threats to species. Threats will be categorized based on the IUCN threat classification scheme categories. Should articles focus on activities that fall under the definition of threat used here but cannot be included in the IUCN categories, they will be assigned to “Others”. A study will be excluded if:
  a. It does not provide information on the link between direct and indirect drivers of biodiversity loss.
  b. It does not analyze human-driven pressures (e.g., natural earthquakes, meteorites).
3. Eligible study type: The literature base will comprise only primary research published between 1999 and 2024 in English, Spanish, French, and Italian. A study will be excluded if:
  c. It is a narrative review with no synthesis of new data.

#### Consistency checking.

Throughout the full-text stage of the screening process, one reviewer will primarily conduct the evaluations. A second reviewer will assess at least 50 papers, or 10% of the total, to ensure consistency, after which Cohen’s Kappa will be calculated. This Kappa analysis will use the categories “Include” and “Exclude” to measure the level of agreement between reviewers [29]. In cases of disagreement, the reviewers will discuss and determine whether to include the articles. If the Kappa statistic is below 0.6, the robustness of the qualifying criteria will be reassessed [31]. It is not anticipated that any reviewer will have authored an article included in the mapping. If such a situation arises, it will be disclosed, along with a detailed rationale for each decision made regarding the contested articles.

#### Study validity assessment.

The quality of included studies will not be evaluated beyond their eligibility based on inclusion criteria. Data categories and study design details will be gathered and coded as indicated in **(****S4 Table****)**.

### Data extraction

Each article included at the full-text stage will be manually coded using a data collection sheet developed by Ridley et al. [16], which will be modified to align with the current research objectives. Key descriptive information regarding the presence of indirect drivers of threats to biodiversity will be examined and coded. Following Díaz et al. [13], each IUCN threat category will be linked to the main groups of direct drivers of biodiversity loss. The data sheet’s usability and clarity were assessed using the test list of fifteen articles (**S2 File**). A second reviewer will code 10% of the papers selected randomly. Any discrepancies will be discussed, and decisions regarding the appropriate coding will be made collaboratively by the reviewers. Data will be collected from articles on bibliographies, study design, direct and indirect threat measurement, ecological levels of organization, and the populations investigated. To be coded, the data must be present in the article or supplementary materials. Authors will not be contacted for missing information.

### Data synthesis and presentation

This study seeks to synthesize information from eligible studies through a systematic mapping exercise. It will provide a comprehensive database of the reviewed literature, along with coded metadata, as supplementary material. We will derive summary statistics from the extracted data and organize them by ecoregion, ecological levels of organization, study scale, and employed methodological approaches. The study will emphasize the presence of indirect drivers of biodiversity loss and their inherent geographic features. We will include detailed information on the progression of articles through the mapping process, specifying the number of papers sourced and those excluded at each stage. We will produce a geographic map to illustrate the global coverage and density of study areas and the distribution of studies across various spatial scales. We will summarize and visualize key extracted information as well as gaps in knowledge about indirect drivers of biodiversity loss and assess relationships among taxonomy, ecological levels of organization, direct and indirect threats studied, and geographic distribution. Finally, we will propose guidelines for future reporting, insofar as permitted by the scope of the extracted data.

## Supporting information

S1 File PRISMA document. (DOCX)

S1 Table ROSES sheet. (XLSX)

S2 File Scoping search for keywords. (DOCX)

S2 Table String search testing and keywords. (XLSX)

S3 Table Full list of benchmark articles. (XLSX)

S4 Table Piloted data extraction strategy. (XLSX)
