# Supplementary material for: What is the evidence on indirect drivers of biodiversity loss worldwide? A systematic map protocol
Source: PLoS One. 2026 Mar 6;21(3):e0341928. doi: 10.1371/journal.pone.0341928 (PMC12965540; doi:10.1371/journal.pone.0341928)
Supplement: S2 File — (DOCX) [file pone.0341928.s003.docx]

# Readme

Supporting File 2: Details of search strategy development including the benchmark list of articles, the scope of the benchmark list, and the identification of keywords.

## 1. Benchmark list of articles

1. Abram, N. K., Meijaard, E., Wells, J. A., Ancrenaz, M., Pellier, A. S., Runting, R. K., ... & Mengersen, K. (2015). Mapping perceptions of species' threats and population trends to inform conservation efforts: the Bornean orangutan case study. *Diversity and Distributions*, *21*(5), 487-499.
2. Birkmanis, C. A., Partridge, J. C., Simmons, L. W., Heupel, M. R., & Sequeira, A. M. (2020). Shark conservation hindered by lack of habitat protection. *Global Ecology and Conservation*, *21*, e00862.
3. Chu, C., Minns, C. K., & Mandrak, N. E. (2003). Comparative regional assessment of factors impacting freshwater fish biodiversity in Canada. Canadian Journal of Fisheries and Aquatic Sciences, 60(5), 624-634.
4. Desta, H., & Fetene, A. (2020). Land-use and land-cover change in Lake Ziway watershed of the Ethiopian Central Rift Valley Region and its environmental impacts. *Land use policy*, *96*, 104682.
5. Di Fulvio, F., Forsell, N., Korosuo, A., Obersteiner, M., & Hellweg, S. (2019). Spatially explicit LCA analysis of biodiversity losses due to different bioenergy policies in the European Union. *Science of the total environment*, *651*, 1505-1516.
6. Enrichetti, F., Bo, M., Morri, C., Montefalcone, M., Toma, M., Bavestrello, G., ... & Bianchi, C. N. (2019). Assessing the environmental status of temperate mesophotic reefs: A new, integrated methodological approach. Ecological Indicators, 102, 218-229.
7. Hoang, N. T., Taherzadeh, O., Ohashi, H., Yonekura, Y., Nishijima, S., Yamabe, M., ... & Kanemoto, K. (2023). Mapping potential conflicts between global agriculture and terrestrial conservation. *Proceedings of the National Academy of Sciences*, *120*(23), e2208376120.
8. Kamino, L. H. Y., Pereira, E. O., & do Carmo, F. F. (2020). Conservation paradox: Large-scale mining waste in protected areas in two global hotspots, southeastern Brazil. Ambio, 49(10), 1629-1638.
9. Micheli, F., Halpern, B. S., Walbridge, S., Ciriaco, S., Ferretti, F., Fraschetti, S., ... & Rosenberg, A. A. (2013). Cumulative human impacts on Mediterranean and Black Sea marine ecosystems: assessing current pressures and opportunities. PloS one, 8(12), e79889.
10. Moran, D., & Kanemoto, K. (2017). Identifying species threat hotspots from global supply chains. *Nature Ecology & Evolution*, *1*(1), 0023.
11. Schulte to Bühne, H., Wegmann, M., Durant, S. M., Ransom, C., de Ornellas, P., Grange, S., ... & Pettorelli, N. (2017). Protection status and national socio‐economic context shape land conversion in and around a key transboundary protected area complex in West Africa. *Remote Sensing in Ecology and Conservation*, *3*(4).
12. Selkoe, K. A., Halpern, B. S., Ebert, C. M., Franklin, E. C., Selig, E. R., Casey, K. S., ... & Toonen, R. J. (2009). A map of human impacts to a “pristine” coral reef ecosystem, the Papahānaumokuākea Marine National Monument. *Coral Reefs*, *28*, 635-650.
13. Sun, Z., Behrens, P., Tukker, A., Bruckner, M., & Scherer, L. (2022). Global human consumption threatens key biodiversity areas. *Environmental Science & Technology*, *56*(12), 9003-9014.
14. Symes, W. S., Edwards, D. P., Miettinen, J., Rheindt, F. E., & Carrasco, L. R. (2018). Combined impacts of deforestation and wildlife trade on tropical biodiversity are severely underestimated. *Nature communications*, *9*(1), 4052.
15. Weinzettel, J., Vačkář, D., & Medková, H. (2018). Human footprint in biodiversity hotspots. *Frontiers in Ecology and the Environment*, *16*(8), 447-452.

## 2. Scope of the benchmark list

| **Authors** | **Spatial Scale** | **Country** | **Ecological level** | **Ecoregion** | **Taxonomic resolution** |
| --- | --- | --- | --- | --- | --- |
| Abram_etal | Local | Indonesia | population | Terrestrial | mammal |
| Birkmanis_etal | National | Australia | population | Marine | fish |
| Chu_etal | National | Canada | biome | Freshwater | fish |
| Desta and Fetene | Local | Ethiopia | ecosystem | Freshwater;Terrestrial | forest |
| Di_Fulvio_etal | Continental | na | biome | Terrestrial | mammal;amphibia;bird;reptile;tracheophyta |
| Enrichetti_etal | National | Italy | biome | Marine | fish |
| Hoang_etal | Global | na | biosphere | Terrestrial | mammal;amphibia;bird;reptile;tracheophyta |
| Kamino_etal | Sub-national | Brazil | ecosystem | Freshwater | other |
| Micheli_etal | Multi-national | Bosnia and Herzegovina; […]; Russia | biome | Marine | na |
| Moran and Kanemoto | Global | na | biosphere | Freshwater;Marine;Terrestrial | animal |
| Selkoe_etal | Sub-national | Hawaii | ecosystem | Marine | invertebrates |
| SchulteToBuehne_etal | Multi-national | Benin;Burkina Faso;Niger | biome | Terrestrial | na |
| Sun_etal | Global | na | biosphere | Terrestrial | mammal;amphibia;bird;reptile;tracheophyta |
| Symes_etal | Sub-national | Indonesia | biome | Terrestrial | bird |
| Weinzettel_etal | Global | na | biosphere | Freshwater;Marine;Terrestrial | na |

## 3. Identification of keywords

| **Article** | **Population** | **Outcome** | **Study type** | **Context** |
| --- | --- | --- | --- | --- |
| ABRAM_etal | population trends | perceptions | **map**ping | **conservation** efforts |
|  | wildlife **species** | **threats** | predicting | management strategies |
|  | endangered | conflicts | local knowledge |  |
|  |  | killings | interview questionnaires |  |
|  |  | **human**-orangutan |  |  |
|  |  | decline |  |  |
| BIRKMANIS_etal | **habitat** | decline | generalised linear models | protection |
|  | shark |  | occurrences | **conservation** |
|  | marine parks |  | predict | management |
|  | protected areas |  |  |  |
|  | suitable **habitat** |  |  |  |
| CHU_etal | freshwater fish | **stress** index | comparative regional assessment | **biodiversity** |
|  | **species** | **human stress**es | environmental index | **conservation** |
|  | richness |  | priority ranking |  |
|  | watershed |  | regional analyses |  |
| DESTA AND FETENE | watershed | land-use | satellite image | measures |
|  | **ecosystems** | land cover change | **map**s | **conservation** interventions |
|  | environmental resources | environmental **impacts** | geographical information systems |  |
|  |  | degradation |  |  |
|  |  | **human pressures** |  |  |
| DI FULVIO_etal | **species** | **loss** | **spatial**ly explicit | **biodiversity** |
|  | biomass | land-use | LCA analysis | bioenergy policies |
|  |  | **footprint** | **impact** assessment |  |
|  |  | demand |  |  |
|  |  | bioenergy |  |  |
| ENRICHETTI_etal | **habitat**s  **ecosystems**  **species** diversity | vulnerable  fishing **pressure**  **anthropogenic** factors | multi-parametric index | environmental status  environmental conditions |
| HOANG_etal | **biodiversity** | potential conflicts | **spatial** analysis | terrestrial **conservation** |
|  |  | land-use | **map**ping | priority |
|  |  | demand | GIS |  |
|  |  | **risk hotspots** |  |  |
|  |  | **threat** |  |  |
| KAMINO_etal | protected areas | large-scale mining waste | **spatial** overlap | **conservation** |
|  | **hotspot**s | damage |  | public policies |
|  |  | **loss**es |  | management and monitoring mechanisms |
|  |  | costs |  |  |
| MICHELI_etal | marine **ecosystems** | cumulative **human impact**s | **spatial** information | management |
|  | ecoregions | **pressure**s |  | protection |
|  |  | **human** activities |  | policy efforts |
| MORAN AND KANEMOTO | **species** | **threat hotspot**s | **spatial**ly explicit | **conservation** |
|  |  | overexploitation | supply chains | measures |
|  |  | consumer demand | **map** | **biodiversity** |
|  |  | **threat**ened | connecting | **action**s |
|  |  | **footprint** | linked |  |
| SCHULTEtoBUEHNE_etal | protected area | land conversion | remote sensing | protection status |
|  | **hotspot** | agricultural expansion |  | transboundary cooperation |
|  |  | rural population growth |  | **biodiversity** |
|  |  | **anthropogenic** **pressure** |  |  |
| SELKOE_etal | **ecosystem** | **human** **impacts** | **map** | marine **conservation** |
|  |  | **anthropogenic threats** | **spatial** data | regional management |
|  |  | cumulative **impacts** | **habitat map**s | surveillance priorities |
|  |  |  | expert judgment |  |
|  |  |  | regional analysis |  |
| SUN_etal | key **biodiversity** areas | **pressure** | global economic model | global **biodiversity** |
|  | plant | **human** activities | **spatial map**ping |  |
|  | vertebrate | land-use | countryside species−area relationship |  |
|  |  | **loss** | multiregional input−output analysis |  |
| SYMES_etal | bird | combined **impacts** | forest extent **maps** | wildlife trade |
|  | endemic **species** | deforestation | exploitation **pressure** | tropical **biodiversity** |
|  | **hotspot** | **habitat loss** |  |  |
|  |  | exploitation |  |  |
| WEINZETTEL_etal | **hotspots** | **human** **footprint** | supply chains | **biodiversity** |
|  | natural **ecosystems** | **direct** factors | **spatial** | **conservation** |
|  | net primary production | **drivers** |  | priority |
|  |  | **ecosystem** change |  |  |
|  |  | **loss** |  |  |
|  |  | consumption |  |  |

*Table 1. Keywords related to the search phrases used to limit search results that are included in the test-set of benchmark articles' titles, abstracts, and keywords. The list of contextually broad terms that must be included for a literature search to retrieve the entire test-set of articles is highlighted in bold.*
